# The Nociceptin/Orphanin FQ Receptor Antagonist UFP-101 Reduces Microvascular Inflammation to Lipopolysaccharide *In Vivo*

**DOI:** 10.1371/journal.pone.0074943

**Published:** 2013-09-23

**Authors:** Zoë L. S. Brookes, Emily N. Stedman, Nicola J. Brown, Christopher P. Hebbes, Remo Guerrini, Girolamo Calo, Charles S. Reilly, David G. Lambert

**Affiliations:** 1 Microcirculation Research Group, Department of Cardiovascular Sciences, University of Sheffield, Sheffield, United Kingdom; 2 Department of Cardiovascular Sciences, Division of Anaesthesia, Critical Care and Pain Management, University of Leicester, Leicester Royal Infirmary, Leicester, United Kingdom; 3 Department of Pharmaceutical Sciences and Biotechnology Center, University of Ferrara, Ferrara, Italy; 4 Department of Experimental and Clinical Medicine, Section of Pharmacology, University of Ferrara and Italian Institute of Neuroscience, Ferrara, Italy; Midwestern University, United States of America

## Abstract

Microvascular inflammation occurs during sepsis and the endogenous opioid-like peptide nociceptin/orphanin FQ (N/OFQ) is known to regulate inflammation. This study aimed to determine the inflammatory role of N/OFQ and its receptor NOP (ORL1) within the microcirculation, along with anti-inflammatory effects of the NOP antagonist UFP-101 (University of Ferrara Peptide-101) in an animal model of sepsis (endotoxemia).

Male Wistar rats (220 to 300 g) were administered lipopolysaccharide (LPS) for 24 h (-24 h, 1 mg kg^-1^; -2 h, 1 mg kg^-1^ i.v., tail vein). They were then either anesthetised for observation of the mesenteric microcirculation using fluorescent *in vivo* microscopy, or isolated arterioles (~200 µm) were studied *in vitro* with pressure myography.

200 nM kg^-1^ fluorescently labelled N/OFQ (FITC-N/OFQ, i.a., mesenteric artery) bound to specific sites on the microvascular endothelium *in vivo*, indicating sparse distribution of NOP receptors. *In vitro*, arterioles (~200 µm) dilated to intraluminal N/OFQ (10^-5^M) (32.6 + 8.4%) and this response was exaggerated with LPS (62.0 +7.9%, *p*=0.031). *In vivo*, LPS induced macromolecular leak of FITC-BSA (0.02 g kg^-1^ i.v.) (LPS: 95.3 (86.7 to 97.9)%, p=0.043) from post-capillary venules (<40 µm) and increased leukocyte rolling as endotoxemia progressed (p=0.027), both being reduced by 150 nmol kg^-1^ UFP-101 (i.v., jugular vein).

Firstly, the rat mesenteric microcirculation expresses NOP receptors and secondly, NOP function (ability to induce dilation) is enhanced with LPS. UFP-101 also reduced microvascular inflammation to endotoxemia *in vivo*. Hence inhibition of the microvascular N/OFQ-NOP pathway may have therapeutic potential during sepsis and warrants further investigation.

## Introduction

The presence of bacteria in the bloodstream, or sepsis, results in generalised inflammation of blood vessels, hypotension and hypovolemia, which together cause tissue damage and poor perfusion of organs, potentially resulting in multiple organ failure. In the United States there are an estimated 20 million annual cases of severe sepsis [1]. With a mortality rate of 35% this suggests around 700,000 deaths per year, exceeding mortality rates for breast and colon cancer [1]. Pharmacological treatments remain inadequate therefore it is of utmost importance to discover new pathways that could be targeted therapeutically during this potentially fatal condition.

Nociceptin/orphanin FQ (N/OFQ) is an opioid-like peptide and an endogenous ligand for the NOP receptor (previously known as ORL1), which is coupled to the G_i_/G_o_ signalling pathway [2]. With the exception of kappa;KOP (very weak) there is no activity of N/OFQ at classical opioid receptors (mu;MOP, delta;DOP or KOP [2]). NOP is expressed on aortic endothelium [3], and following intravenous (i.v.) and intracerebroventricular (i.c.v.) administration, N/OFQ caused both hypotension and bradycardia [4-6]. N/OFQ also increased aortic blood flow and induced dilation of large blood vessels >200 µm [4,7]. The cardiovascular effects of N/OFQ are absent in mouse knockouts for NOP (NOP^-/-^) [8]. Within smaller blood vessels (15 to 40 µm) N/OFQ also induces dilation, along with inflammatory responses such as increased permeability and leukocyte-endothelial interactions within postcapillary venules [9]. Furthermore, white blood cells express NOP [10,11], but NOP expression on the microvascular endothelium has not yet been directly identified. Our preliminary experiments using qPCR suggested that protein levels of NOP within the microcirculation may be too low for analysis of expression via this technique. Hence we labelled N/OFQ with a fluorescent marker (fluoroscein isothiocyanate, FITC) to produce FITC-N/OFQ, which could then be injected locally at high concentrations to identify the distribution of NOP in small vessels.

The N/OFQ-NOP pathway may also play a role in sepsis [12], with plasma N/OFQ concentrations being higher in septic patients who died within 30 days, compared to survivors [11]. Upregulation of N/OFQ has also been reported in rat sensory neurones and hypothalamus in response to lipopolysaccharide (LPS), a component of gram negative bacteria, with antibodies to the receptor for LPS (toll-like receptor-4; TLR-4) also preventing induction of N/OFQ by LPS in murine dorsal root ganglion neurons [13-15]. Nevertheless, studies using isolated rat splenocyte cells did not detect significantly increased levels of N/OFQ released from cells until 18 hours of stimulation with LPS [16]. For this reason, combined with the fact that many patients are not treated until sepsis is well established, we studied N/OFQ 20 to 24 hours after the onset of endotoxemia.

Thus far N/OFQ (0.1 mg kg^-1^, s.c.) has been found to decrease survival from 30% to 0% at 5 days in the cecal ligation and puncture (CLP) model of sepsis in rats [17]. In conjunction with proposed inflammatory role for N/OFQ [2], we further hypothesised that the function of microvascular NOP-N/OFQ would be altered in an established LPS model of sepsis (endotoxemia) [18,19].

Should the NOP-N/OFQ pathway be upregulated during endotoxemia one would anticipate that manipulating this pathway could be of therapeutic benefit. Only one study has previously utilised the selective NOP antagonist [Nphe^1^, Arg^14^,Lys^15^] N/OFQ-NH_2_ (UFP-101) [20] during sepsis: in rats, Carvalho et al. demonstrated that 0.03 mg kg^-1^ UFP-101 improved survival rates from approximately 30% to 65%. 0.03 mg kg^-1^ UFP-101 also reduced plasma concentrations of TNF-α and IL-1β in response to CLP [17], and when given i.c.v. reduced the hypothalamic pituitary adrenal stress response to LPS [14]. The current study was important to determine whether UFP-101 was similarly effective in the LPS model of sepsis, and whether beneficial effects were reproduced at the microvascular level.

This study aimed to determine the inflammatory role of N/OFQ and its receptor NOP (ORL1) within the microcirculation, along with anti-inflammatory effects of the NOP antagonist UFP-101 (University of Ferfara Peptide-101) in an animal model of sepsis (endotoxemia).

## Materials and Methods

### Animals and Housing

Male Wistar rats (200 to 300 g; Harlan, UK, *n*=47) were obtained from the Field Laboratories at the University of Sheffield, UK. All experiments were performed ethically in accordance with the UK Home Office, Project Licence number 40/2809. Animals were held in the animal facilities for at least 1 week before experimental procedures, exposed to light on a 12: 12 h cycle in a humidity- and temperature-controlled environment, maintained on 0.3% sodium, standard, pelleted, commercial diet and allowed water *ad libitum*.

### Drugs and solutions

The constituents of the HEPES-buffered phosphate saline (HEPES-PBS) solution were: 0.2884 g l^-1^ MgSO_4,_ 0.245 g l^-1^ CaCl_2,_ 2.383 g l^-1^ HEPES, 8.2983 g l^-1^ NaCl, 0.3504 g l^-1^ KCl, 0.1606 g l^-1^ KH_2_PO_4_. On the day of use 0.99 g l^-1^ of D(+) glucose was added to the solution and the pH adjusted to 7.4 [21]. Fluoroscein isothiocyanate (FITC) (10%, A1628, Sigma, UK) was conjugated to bovine serum albumin (66 KDa BSA (98%), A7030, Sigma) as previously described [22], at 5°C to avoid denaturing the BSA. N/OFQ and UFP101 were also prepared in house as previously described [22,23]. All other drugs were obtained from Sigma unless stated.

### Synthesis of [Lys(FITC)^18^]N/OFQ-NH_2_ (FITC-N/OFQ)

Synthesis of FITC-N/OFQ (MW: 2895) was performed using the intermediate [Lys(Dde)^18^] N/OFQ-NH_2_ in accordance with procedures previously reported [23]. Selective deprotection of the Lys^18^ side chain was achieved by following the procedure of Bycroft et al. [24].

300 mg of protected [Lys(Dde)^18^] N/OFQ-NH_2_ -resin suspended in anhydrous tetrahydrofuran (THF, 3 ml) was added to 2% hydrazine in dimethylformamide (DMF, 5 ml). The reaction mixture was stirred under argon for 30 min, the solution filtered and the resin washed 3 times with DMF (5 ml) and CH_2_Cl_2_ (5 ml). To a suspension of protected [Lys(free side chain)^18^] N/OFQ-NH_2_-resin (170 mg, 0.7 meq/gr; 0.12 meq) in freshly distilled tetrahydrofuran (THF, 3 ml), fluorescein isothiocyanate isomer I (FITC) (0.2 meq) in THF : absolute Et-OH (1 : 1, 3 ml) and triethylamine (0.25 ml) was then added. The solution was stirred in the dark under argon at room temperature for 12 h. The solution was then filtered and the resin washed 5 times with THF (5 ml) and 3 times with CH_2_Cl_2_ (5 ml). The protected [Lys(FITC)^18^] N/OFQ-NH_2_-resin was treated with reagent B [25], which contained trifluoroacetic acid (TFA) : H20 : phenol : triisopropylsilane (88 : 5 : 5: 2 v/v; using 10 ml per 0.2 g of resin) for 1.5 h at room temperature in the dark. After filtration of the resin, the solvent was concentrated under vacuum and the residue triturated with ether. The crude peptide was purified by preparative reverse phase HPLC and lyophilized.

### Receptor and GTPγ[^35^S] binding to recombinant opioid and NOP receptor

Experiments were performed using Chinese hamster ovary (CHO) cells stably expressing human recombinant MOP, DOP, KOP and NOP receptor. Selectivity of binding was assessed by measuring the displacement of [^3^H] diprenorphine ([^3^H]DPN) from membranes prepared from CHO cells expressing MOP, DOP and KOP or [^3^H] N/OFQ to membranes prepared from CHO cells expressing NOP receptors. Endomorphin-1, Leu-enkephalin, norbinaltorphimine and N/OFQ were included as reference ligands for MOP, DOP, KOP and NOP respectively. Data are expressed as % displacement of bound radioligand by FITC-N/OFQ and the reference compounds. The concentration of displacer producing 50% displacement of specific binding was corrected for the competing mass of radioligand according to Cheng and Prusoff to yield pK_i_ [26], a measure of affinity. Functional activity of FITC-N/OFQ was assessed by measuring agonist stimulated GTPγ[^35^S] binding in membranes prepared from CHO cells expressing NOP receptors [27]. The concentration of ligand (FITC-N/OFQ or N/OFQ) producing 50% of the maximum response (E_max_, efficacy) is a measure of potency (EC_50_). All analysis was performed using GraphPad Prizm V4.0.

### Intravital microscopy (IVM)

#### Surgery

Rats were anesthetised with thiopental (induction, 30 mg kg^-1^; maintenance, 40-90 mg kg^-1^ h^-1^; Intra-Vital Sodium, Rhone-Poulenc Rourer, West Malina, UK) to prepare the mesentery for fluorescent IVM [9]. The right jugular vein was cannulated for administration of FITC-BSA and the left carotid artery was cannulated for computerised recording of mean arterial pressure (MAP) using WINDAQ (DI-400, DATAQ Instruments, Akron, OH, USA).

#### Image analysis

Images were monitored using a CCD camera (TK-C13060B, JVC, UK) displayed on a high-resolution monitor (PVM-14N5MDE, Sony, UK) and recorded on to CD-RW using a DataVideo^TM^ CD recorder (VDR-3000, Holdan Ltd, UK) for later off-line computerised image analysis (Capiscope^TM^, KK Technology, UK) as previously described [19]. Prior to experimentation a 30 minute equilibration period was allowed (T_-60_-T_-30_), during which time one area of interest containing an arteriole (15 to 40 µm) and venule (25 to 70 µm) was selected with transmitted light for the purposes of image analysis. At T_-30_ FITC-BSA (IVM, Study B) also was given via the jugular vein (0.2 g kg^-1^) to allow fluorescent images to be obtained every 10 minutes during experimentation. At each time point the maximum exposure to blue light (460 to 490 nM) was <60 seconds to prevent photoactivation and tissue damage [28].

To measure *receptor binding* or *macromolecular leak* Capiscope^TM^ assigned an integer value to the brightness of the fluorescence based on an 8-bit arbitrary gray scale ranging from 0 (black) to 255 (white), with fixed brightness and contrast levels. Receptor binding (IVM, Study A) was assessed in each animal by using Capiscope to place a box at three sites where ‘hot-spots’ of receptor binding could be seen. Macromolecular leak (Study B) was measured at three points within the interstitium adjacent (<2 mm) to a randomly selected postcapillary venule, Rolling leukocytes were assessed within a relatively straight length of vessel, using transmitted light, by counting the numbers that passed a fixed point over 30 seconds [9].

#### Experimental groups

In study A the mesenteric artery was also cannulated to administer FITC-N/OFQ (200 nM kg^-1^, 40 nM) intra-arterially at T_0_ (n=5). Images of the microcirculation were then recorded continuously for 60 seconds, which was the duration of visible fluorescence. At the following time point (T_-15_), unlabelled N/OFQ (200 nM kg^-1^, 40nM) was administered 15 seconds before 200 nM kg^-1^ FITC-N/OFQ in order to occupy the NOP receptor site.

In study B animals received LPS (Serotype B5: 055) or the equivalent volume of saline (0.1ml 100g^-1^) into the tail vein at -24 h (1 mg kg^-1^) and -2 h (0.5 mg kg^-1^) prior to experimentation (T_0_) in (i) control, n=6; (ii) LPS, n=6, (iii) UFP-101, n=6 and (iv) LPS + UFP-101, n=6 groups. Animals received UFP-101 (150 nM kg^-1^, 62.5nM), or the equivalent volume of saline (1 ml kg^-1^) into the jugular vein at the baseline recording (T_0_). Images of the microcirculation were recorded for 60 seconds with transmitted light and 30 seconds with blue light (460-490nM) every 10 minutes at T_10_, T_20_, T_30_ and T_40_. A full response curve was thus constructed, but data are shown graphically only at T_40_ for ease of interpretation as similar patterns of response were observed at earlier time points.

At the end of the procedure rats were killed humanely in accordance with UK Home Office procedure using an overdose of anesthetic followed by cervical dislocation.

### Pressure myography

#### Surgery

Rats were killed in accordance with UK Home Office requirements involving cervical dislocation. This was followed by rapid removal of the ileum and adjacent mesentery from either control or LPS-treated animals. The mesentery was placed in HEPES-PBS solution at 4°C and a third order artery (~200µm) dissected free from surrounding adipose tissue. Vessels were carefully transferred to the organ bath and mounted on glass cannulae at 60mmHg (Living Systems Instrumentation, Burlington, Vermont, USA). Vessels were stabilised and maintained as previously described, with the Living Systems video dimension analyser used to determine luminal diameters^21^. In previous studies it has been determined that rat mesenteric vessels do not respond to abluminal N/OFQ, hence N/OFQ was administered intraluminally using pre-determined doses [9].

#### Experimental groups

Animals were divided into either (i) control (*n* = 6); (ii) N/OFQ (*n* = 6) or (iii) LPS + N/OFQ (*n* = 6) treated groups. LPS was administered into the tail vein at -24 h (1mg kg^-1^) and -2 h (0.5mg kg^-1^) (iii), or the equivalent volume of saline (1ml kg^-1^) (i, iii), and then mesenteric tissue removed after 24 h of endotoxemia. Vessels were also exposed to 50 µg ml^-1^ LPS (Serotype B5: 055) in the organ bath for the duration of myography.

After performing a concentration response curve with U46619 (10^-10^ to 10^-6^M), all vessels were pre-constricted with an EC_80_ of U46619 (2x10^-7^M). U46619 is synthetic analogue of prostaglandin H_2_ which acts as a thromboxane A_2_ receptor agonist and commonly used in myography to pre-constrict isolated vessels before studying vasodilator responses. Thus following pre-constriction at a constant pressure (60 mmHg) HEPES (i, iii) or the calculated EC_80_ dose of N/OFQ (10^-5^M) (ii) was then allowed to flow through the lumen of pre-constricted vessels for 20 minutes at a flow rate of 15 µl min^-1^ [9]. Measurements of luminal diameter were taken every minute, but data are shown such that the percentage change in the diameters of vessels is reported between T_0_ and T_20_. At the end of the protocol, the organ bath was washed out and acetylcholine (ACh, 10^-5^M) was added to pre-constricted vessels to ensure that endothelial integrity had been maintained.

### Statistics

Parametric data are presented as mean (+SEM), with statistical analysis performed using two-way ANOVA or student *t*-test. However, all *in vivo* data were non-parametric and presented as median and interquartile ranges (25^th^ to 75^th^), with *n* = 5 or 6 animals used in each group, as determined using preliminary data. Data are expressed as box and whisker plots, except where negative values were present within data and this was not possible. In study A paired data were analysed using the Wilcoxon paired signed rank test at equivalent time points. In study B, if Kruskall-Wallis test identified significant differences between groups, analyses was performed using the Mann-Whitney *U*-test. Likewise, comparing data to baseline, if the Freidman test identified a significant difference then the Wilcoxon signed ranks test was performed. To avoid significance being obtained due to multiple tests, only one time point underwent statistical evaluation using post-hoc tests. All statistical analysis was undertaken by an independent statistician using SPSS version 16.0. Results were considered statistically significant at *p* < 0.05.

## Results

### Preliminary characterisation of FITC-N/OFQ

**Figure 1:**
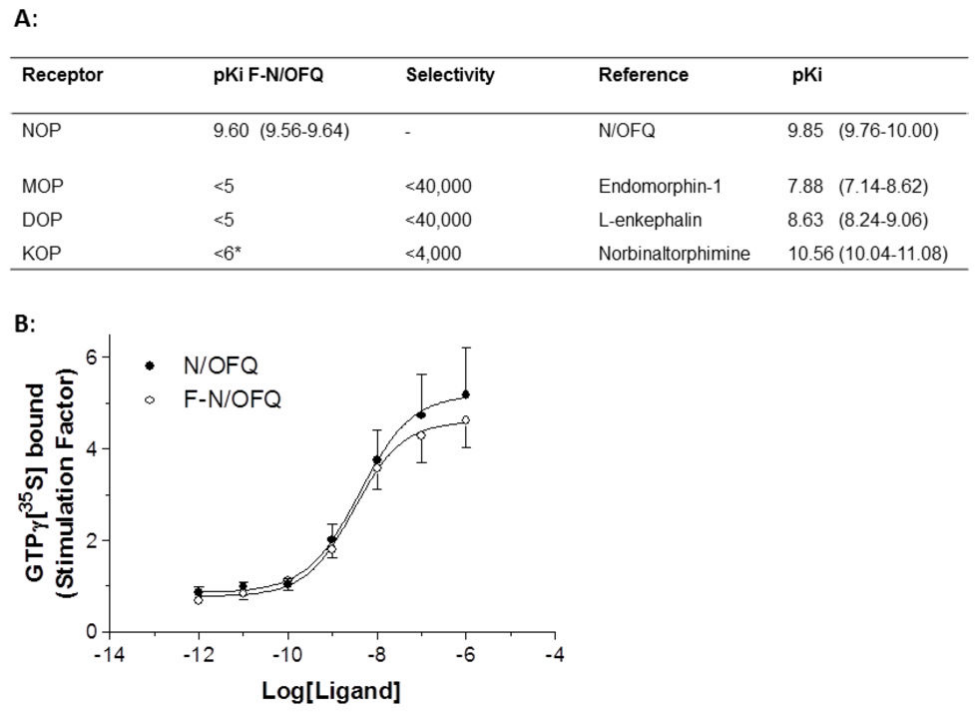
Characterisation of FITC-N/OFQ (F-N/OFQ) at recombinant NOP and classical opioid receptors on CHO cells. **A**: Binding affinity of F-N/OFQ and a range of opioid receptor subtype selective reference compounds. F-N/OFQ shows high selectivity for NOP over classical opioid receptors (mean (range) of up to 4 experiments.) *curves did not saturate K_i_ estimated between 1 and 10µM. **B**: Both N/OFQ and F-N/OFQ behave as full agonists on recombinant human NOP. These data are stimulation factor = agonist stimulated specific binding / basal specific binding (mean +SEM, *n*=8).

In receptor binding experiments FITC-N/OFQ showed high affinity (pK_i_ 9.60 compared to 9.85 for N/OFQ) for NOP and at least 4000 fold selectivity over KOP (Figure 1A). As anticipated the high affinity binding of FITC-N/OFQ resulted in a high potency stimulation of the binding of GTPγ[^35^S] to membranes prepared from cells expressing NOP (Figure 1B). There was no difference in potency (pEC_50_ ~8.4) or efficacy (E_max_: 4.7 to 5.2 expressed as stimulation factor) between the native peptide and FITC-N/OFQ. Based on this simple *in vitro* characterisation FITC-N/OFQ does not differ substantially from native N/OFQ in terms of affinity, selectivity, functional potency and efficacy indicating its suitability for use in the *in vivo* experiments described below.

### Evidence for N/OFQ specific binding within mesenteric microcirculation

**Figure 2:**
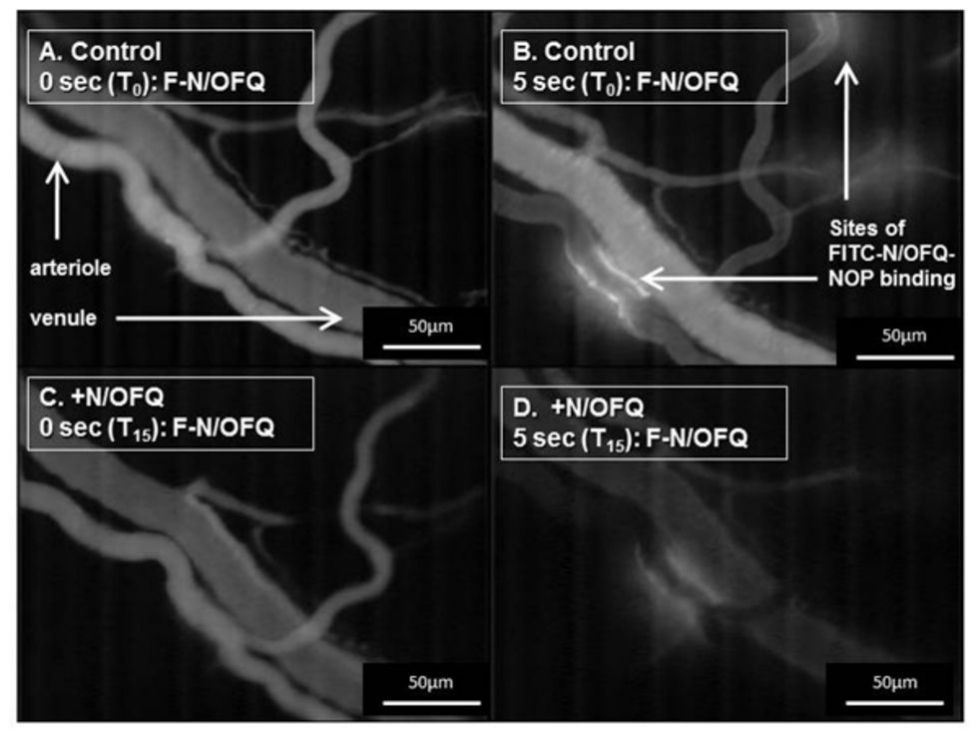
Representative images of rat mesenteric arterioles and venules 5 seconds after injection of 200 nM kg^-1^ FITC-N/OFQ into the mesenteric artery with (C,D) and without (A,B) N/OFQ (added 15 sec prior to FITC-N/OFQ and labelled T_15_) competing for the same NOP receptor site. The areas highlighted by arrows indicate binding of FITC-N/OFQ to NOP (FITC-N/OFQ-NOP) and thus sparse distribution of NOP receptors on the endothelium in this animal.

**Figure 3:**
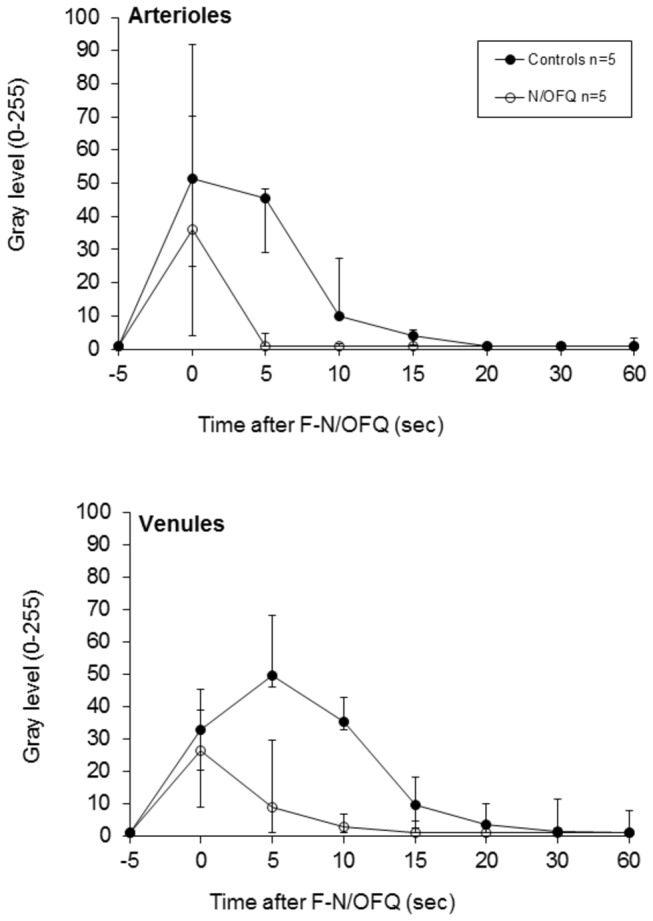
The decay of FITC fluorescence (gray level) in the endothelium of rat mesenteric arterioles and venules over 60 seconds in response to 200 nM kg^-1^ FITC-N/OFQ (F-N/OFQ), with (T-_15_, open circles) and without (T_0_, closed circles) 200nM.kg^-1^ N/OFQ (n=5 animals). Values are median, with upper and lower error bars representing the 75^th^ and 25^th^ percentiles respectively. The time courses were significantly different.

*In vivo*, FITC-N/OFQ bound to specific sites on the endothelium of arterioles, indicating sparse distribution of NOP receptors in the mesentery in control conditions (Figure 2). This binding appeared to be specific, as N/OFQ prior to FITC-N/OFQ (competing for the same receptor site) reduced the the ability to bind at these sites (Figures 2 and 3).

### Cardiovascular and microvascular effects of N/OFQ

*In vivo*, dose dependent hypotension, dilation of mesenteric arterioles and venules, along with macromolecular leak were observed with N/OFQ in agreement with previous studies (data not presented)^9^. In addition, pressure myography demonstrated that N/OFQ caused isolated arterioles to dilate by 32.6 +8.4%, compared to only 6.8 +3.8% in controls (*p*=0.031).

### Altered responses to N/OFQ during LPS administration

#### Cardiovascular

**Figure 4:**
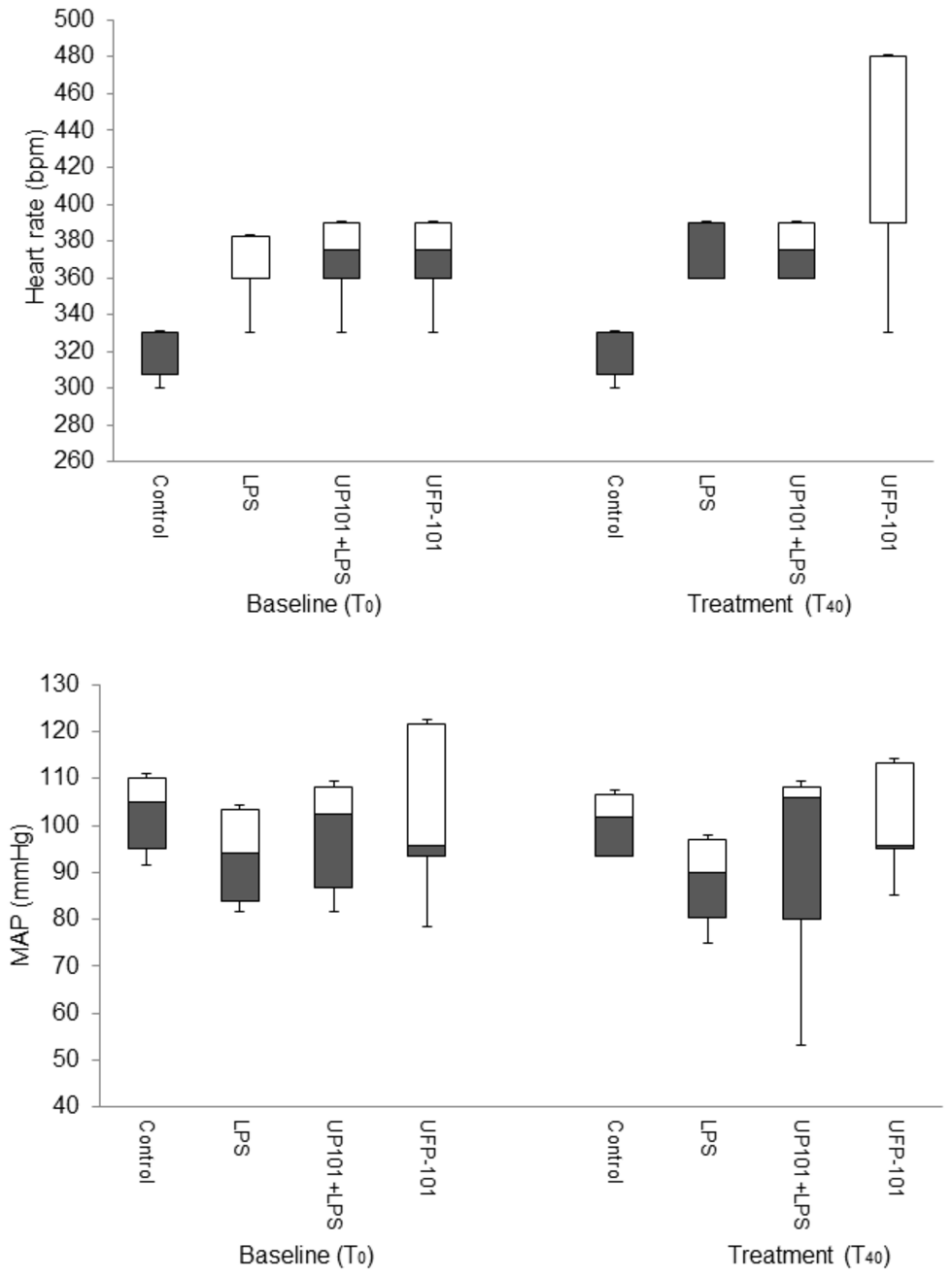
The effect of UFP-101 on cardiovascular variables: mean arterial pressure (MAP, mmHg; upper panel) and heart rate (beats per minute, bpm; lower panel) were measured in anesthetised rats at baseline (T_0_), this being 24 hours after i.v. injection with 1 mg kg^-1^ + 0.5 mg kg^-1^ (LPS, n=6; LPS + UFP-101, n=6) or saline (control, n=6; UFP-101, n=6). Measurements were repeated 40 minutes after baseline (T_40_) in response to i.v. injection of 150 nM kg^-1^ UFP-101 (LPS + UFP-101, UFP-101 groups) or saline (control; LPS, groups). Values are median, with open bars representing the 50^th^-75^th^ percentile, grey bars the 25^th^-50^th^ percentile and upper and lower error bars representing the maximum and minimum respectively.

LPS caused a significant increase in heart rate (T_40_, *p*=0.004), but this was not altered by the addition of UFP-101 (*p*=0.004, Figure 4). LPS also caused hypotension compared to controls (T_40_, control: 105 (95 to 110) mmHg; LPS: 85 (83 to 103) mmHg) (Figure 4), but this did not reach significance (*p*=0.429).

UFP-101 alone also caused an increase in heart rate (T_40_, control: 330 (308 to 330) bpm, UFP-101: 375 (360 to 390) bpm) (Figure 4, *p*=0.04), but had no significant effect on MAP.

#### Microvascular

Pressure myography demonstrated that LPS enhanced dilation of isolated arterioles to 10^-5^M N/OFQ, as arterioles in the N/OFQ + LPS group dilated by 62.0 +7.9% compared to 32.6 +8.4% with N/OFQ alone (*p*=0.031).

**Figure 5:**
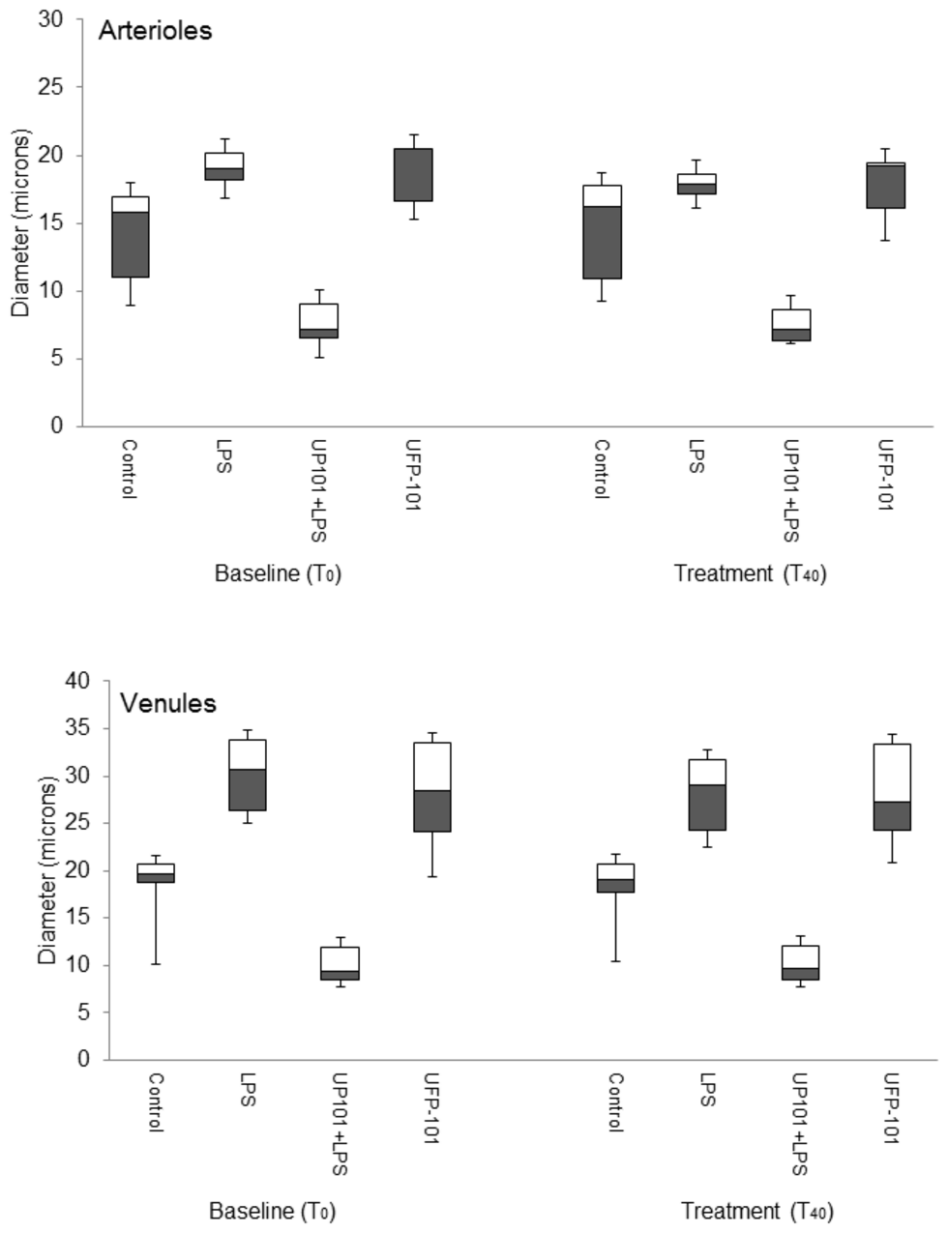
The effect of UFP101 on microvessel diameters *in vivo*: arteriole (upper panel) and venule (lower panel) diameters were measured in anesthetised rats at baseline (T_0_), this being 24 hours after i.v. injection with 1 mg kg^-1^ + 0.5 mg kg^-1^ (LPS, n=6; LPS + UFP-101, n=6) or saline (control, n=6; UFP-101, n=6). Measurements were repeated 40 minutes after baseline (T_40_) in response to i.v. injection of 150 nM kg^-1^ UFP-101 (LPS + UFP-101, UFP-101 groups) or saline (control; LPS, groups). Values are median, with open bars representing the 50^th^-75^th^ percentile, grey bars the 25^th^-50^th^ percentile and upper and lower error bars representing the maximum and minimum respectively.

Venules appeared dilated slightly in LPS-treated animals compared to controls (T_40_, control: 19.1 (17.8 to 20.8) µm, LPS: 29.1 (24.2 to 31.8) µm) (Figure 5, *p*=0.002), but diameter changes in arterioles were not significant (*p*=0.057) (Figure 5).

**Figure 6:**
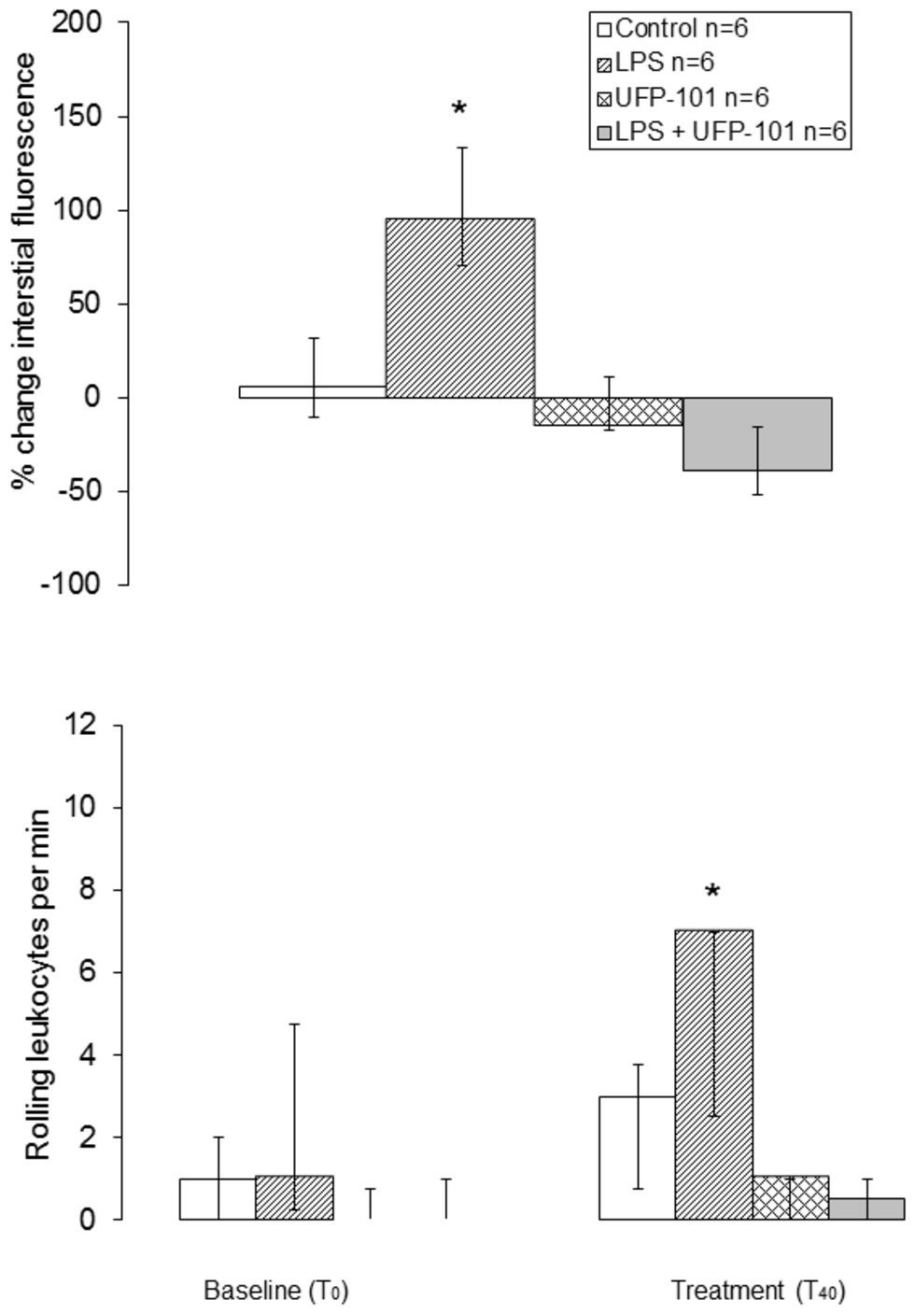
The effect of UFP-101 on macromolecular leak and leukocyte rolling *in vivo*: macromolecular leak (percentage change in interstitial FITC-BSA fluorescence from baseline, upper panel) and leukocyte rolling (per minute, baseline and after treatment, lower panel) in post capillary venules (<40 µm) within the anesthetised rat mesentery preparation. Measurements were taken in response to i.v. injection of 150 nM kg^-1^ UFP-101 (LPS + UFP-101 and UFP-101 groups) or saline (control and LPS groups). Values are median, with open bars representing the 50^th^-75^th^ percentile, grey bars the 25^th^-50^th^ percentile and upper and lower error bars representing the maximum and minimum respectively. ^*^increased compared to control.

LPS caused significant increases in both macromolecular leak and leukocyte rolling (leak, p=0.043; rolling, p=0.027), that were significantly different from controls (T_40,_ leak: controls, 5.3 (-5.9 to 11.8) *versus* LPS: 95.3 (86.7 to 97.9)%, *p*=0.008 and rolling: controls, 3.0 (0.8 to 3.8) *versus* LPS: 7.0 (4.8 to 10.8) per min, *p*=0.003). Macromolecular leak and leukocyte rolling to LPS were reduced by co-administration of 150nmol.kg^-1^ UFP-101 (T_40_, leak: -34.9 (-63.2 to -15.1)% and rolling: 0 (0 to 1) per min), such that they were no longer significantly different from controls (leak, *p*=0.063; rolling, *p*=0.117) (Figure 6).

With UFP101 arterioles (T_40_, controls: 16.2 (10.9 to 17.7) µm; UFP-101: 7.1 (6.3 to 8.6) µm) (*p*=0.004) and venules (controls, 19.1 (17.8 to 20.8) µm; UFP-101: 9.7 (8.4 to 12.1) µm) (*p*=0.001) (Figure 5) appeared constricted compared to controls however, differences between controls and UFP-101 groups were also observed initially between the groups (T_40_, arterioles: *p*=0.004; venules: *p*=0.001). UFP-101 had no effect on either macromolecular leak or leukocyte rolling (leak, *p*=0.151; rolling *p*=0.394).

## Discussion

This study has demonstrated that UFP-101 exhibits anti-inflammatory properties within the *microcirculation*, specifically it reduced macromolecular leak and leukocyte rolling, this potentially being an important contributor to reduced mortality in septic animals treated with UFP-101^17^. Increased blood volume is the basis of current therapeutic strategies, and achieving this via reducing microvessel permeability, in turn increasing blood flow and organ perfusion, confirms promising therapeutic potential for NOP antagonists.

Using a range of complementary experiments and a novel fluorescent analogue of N/OFQ our data also suggested that NOP receptors are expressed on small foci of microvascular endothelium of rat mesentery. This is in agreement with expression of NOP previously reported on the endothelium of larger blood vessels, namely rat aorta [3]. Nevertheless, the present data suggest that expression may be quite sparse in these small vessels. This has functional consequences for NOP partial agonists as they may then behave as antagonists [29]. In conjunction with previous studies, identification of NOP receptors within the microcirculation was an important foundation for understanding how UFP-101 exerted beneficial effects during endotoxemia.

Perhaps the most important part of the study was the therapeutic potential of UFP-101 in an LPS model of sepsis. In agreement with previous observations, that UFP-101 was anti-inflammatory during CLP [17], we also determined that UFP-101 reduced macromolecular leak and reduced leukocyte rolling at the later time point in response to LPS. Furthermore, UFP-101 reduced vasodilation of venules in response to LPS, often associated with excessive production of pro-inflammatory cytokines. Effects on arterioles, which regulate blood pressure, were not observed, but mesenteric venules did respond to LPS in agreement with our previous studies at 0 to 4 h [30]. This may be a compensatory mechanism to preserve blood flow to a vital organ. In conjunction with known reductions in pro-inflammatory cytokines, the ability of UFP-101 to reduce microvascular inflammation may relate to the beneficial effects on mortality during CLP [17]. In agreement, there are increased plasma levels of N/OFQ in patients who die from sepsis *versus* those who do not [31]. Moreover in a very recent study tracking the time course of sepsis in patients on the intensive care unit we have data demonstrating an increase in plasma N/OFQ levels that falls when the patients recover (in this context patients are their own controls) (Thompson, Serrano-Gomez, McDonald, Ladak, Bowrey and Lambert, 2013, unpublished). Of note however, our animal model of sepsis was not particularly severe, as all animals survived the duration of the experiment with hypotension <20%. Whilst non-lethal endotoxin models of sepsis reflect more accurately the insidious onset of the disease, ultimately septicemia is a disease involving the entry of live bacteria, such as *E. Coli* into the bloodstream. In CLP models which reflect this, mortality rates are high: for example the study by Carvalho et al. demonstrated a 70% mortality rate [17]. It is thus re-assuring that these present data with LPS are in agreement with this study. With i.v. administration of UFP-101 we also discovered no reversal of the tachycardia or mild hypotension induced by LPS. This finding is in agreement with direct NOP-N/OFQ inflammatory effects on the microcirculation, but not blood pressure, being a locally mediated mechanism involving histamine [9].

In addition to macromolecular leak, dilation of isolated arterioles (~200µm), was certainly increased by exposure to LPS. This suggests that the function of NOP is enhanced during exposure to LPS; further studies are required to confirm this in smaller vessels *in vivo* and determine the mechanism by which LPS increases NOP expression and/or intracellular signalling. We could not repeat the FITC-N/OFQ experiments in LPS-treated animals, as macromolecular leak interfered with the fluorescent measurements. Interestingly our initial attempts with immunohistochemistry to reproduce the study performed by Granata et al. on the aortic endothelium were unsuccessful using sections of mesenteric microcirculation [3]. This may be because expression is sparse (Figure 2) and radioligand binding studies with [^3^H] UFP-101 are complicated by low radioligand specific activity [32].

To develop this point further, a major criticism of our work might be that we have not demonstrated the presence of NOP using more conventional techniques; (i) radioligand binding and (ii) Western blotting. Due to the very small amount of tissue that can be harvested from the vasculature and the presumed ultra-low expression it was not possible to perform a standard binding experiment of the type shown in Figure 1, even if we employed a high specific activity radioligand ([^125^I] N/OFQ) [33]. The amount of tissue may have been sufficient for Western blotting but, as with most members of the opioid family, there are no selective antibodies for use [34]. In the absence of these, our functional data provides strong evidence for functional NOP and, to reiterate, the completely novel approach used in our present work (FITC-N/OFQ) demonstrated sparse expression in the microvasculature which would not have been possible by other means.

One further limitation of this study is that control animals may have experienced a degree of unavoidable inflammation at baseline, due to the surgery required for observation of the microcirculation using intravital microscopy. In addition, our cardiovascular (and microvascular variables) differed at baseline in all treated groups because animals were already ‘septic’. Indeed, an important limitation of studies using anesthesia/surgery and IVM to observe the mesenteric microcirculation, is that it is not possible to integrate a non-septic baseline or an unanesthetised control. Nevertheless, if there was some degree of inflammation at baseline due to surgery, these data demonstrated that it was reduced in the presence of UFP-101.

Despite the interesting data with inflammatory markers, we could not make firm conclusions as to whether intravenously administered UFP-101 given to non-septic animals truly caused microvascular constriction, as we are uncertain whether this observation was due to differences in the diameter of vessels at baseline or an immediate effect of UFP-101. Despite this, as part unpublished data within previous studies we also observed vasoconstriction with UFP-101 [9]. Compared to larger blood vessels the mesenteric microcirculation as studied here (<40 µm) receives little neural control [35]. Thus, regardless of the baseline status vascular diameters a NOP dependent (rather than neural) mechanism must be engaged for constriction to occur via UFP-101. Taken together with our data utilising isolated vessels and N/OFQ, it is possible that NOP receptors may regulate vessel diameters via endothelial as well as central/neural control mechanisms, as we have alluded to in previous studies proposing an endothelial NOP-histamine pathway [9].

In this study intra-arterially administered UFP-101 had no effect on MAP, similar to studies whereby UFP-101 was given i.c.v. to mice [36]. Further studies likewise reported no differences in MAP between NOP^-/-^ and wild-type mice [8]. However, our findings of tachycardia are in contrast to Burmeister & Kapusta [36]. Nevertheless, similar to our data, 300 pmol UFP-101 injected into the paraventricular nucleus (PVN) of the hypothalamus caused increased heart rate and renal sympathetic nerve activity in conscious Sprague-Dawley rats, along with a non-significant increase in MAP [37]. In our previous studies we did not report the effects of UFP-101 over time, rather its effects on cardiovascular response to N/OFQ [9]. Thus in conjunction with the literature, our new evidence with UFP-101 suggests that the vascular mechanisms of NOP are multifactorial, differing between species and the organ of interest. N/OFQ may also have opposing effects depending upon the route via which it is administered.

At a cellular level activation of NOP will reduce cAMP formation, close voltage gated Ca^2+^ channels and open K^+^ channels to produce hyperpolarisation. In a neurone this combination of events is easy to collate into an inhibition of neurotransmission. In addition to these classical signalling events there is also evidence of increased MAP kinase activity [2]. We do not know how these events transfer to the vascular endothelium, but a modulation of the driving force for dilation (increased Ca^2+^, NO activation or cAMP generation) would seem likely targets and these will need rigorous experimental verification in further studies.

In conclusion, we have evidence that the endothelium of the rat mesenteric microcirculation expresses NOP receptors. Furthermore, in agreement with previous studies using a cecal ligation model, UFP-101, a selective antagonist of NOP, reduces microvascular inflammation during an LPS model of sepsis in rats. Animal studies with small data sets should be considered as preliminary data and thus clinical studies would now be useful to determine if this potentially beneficial therapeutic effect could translate into humans.
